# Audit and Re-audit on Inpatient Prescription Writing in Psychiatry Ward of Allied Hospital 2 Faisalabad

**DOI:** 10.1192/bjo.2025.10576

**Published:** 2025-06-20

**Authors:** Duaa Fatima, Imtiaz Ahmad Dogar, Palvisha Sajid, Sammar Fatima, Sadaf Ghulam Jelani

**Affiliations:** Faisalabad Medical University, Faisalabad, Pakistan

## Abstract

**Aims:** Writing a prescription is an essential task in wards for optimal management of patients. Errors in prescription writing can lead to discrepancy in patient’s care. The aim of this audit is to identify errors in prescription writing in wards, communicate them to the staff for improving standard and quality of care, making sure the provision of adequate medication to the patient.

**Methods:** The study, conducted in early 2023 at a tertiary care hospital in Faisalabad, assessed prescription writing quality through a baseline audit, a faculty-led workshop intervention, and a re-audit after three months. Two team members evaluated randomly selected prescription cards using an audit tool. Results, discussed in a departmental meeting, revealed unsatisfactory standards. In response, mandatory teaching sessions addressed issues like inconsistent expectations, time management, and resident education gaps. Residents were trained on proper prescription documentation and its clinical importance. A follow-up audit showed improvements, highlighting the effectiveness of targeted educational interventions.

**Results:** Prescription charts of patients admitted in psychiatry ward were analysed, which included 156 charts and added up to a total of 624 drug prescriptions in the initial audit which showed severe discrepancies in writing of important parameters. A re-audit was done several months later in which inpatient charts of patients admitted in psychiatry ward were checked, including 200 charts with total 650 prescriptions which indicated positive response from staff resulting in much improvement in writing adequate prescriptions according to defined parameters.

**Conclusion:** This audit suggests the importance of improving prescription writing as 1st audit indicated many errors in prescription writing including missing medicine dosage, frequency or strengths and starting/ending dates of medicines. However in re-audit many improvements were noted in prescription writing in all the highlighted areas. Prescribing errors can be effectively prevented by better education and training of prescribing staff, appropriate task and role definitions, proper supervision and teamwork. After writing a prescription, success of the treatment should be reviewed and proper arrangements must be in place for future review and monitoring.

